# MIS lateral retroperitoneal transpsoas approach allows gross total resection of a giant L4 schwannoma

**DOI:** 10.3171/2022.3.FOCVID2220

**Published:** 2022-07-01

**Authors:** Andres Ramos-Fresnedo, Carlos Perez-Vega, Krishnan Ravindran, W. Christopher Fox

**Affiliations:** Department of Neurosurgery, Mayo Clinic, Jacksonville, Florida

**Keywords:** lateral access spine, lateral spine, lumbar, minimally invasive spine

## Abstract

In this surgical video, the authors present a successful minimally invasive (MIS) lateral retroperitoneal transpsoas approach for resection of an L4 nerve root schwannoma. They describe the surgical approach in detail, with special emphasis on patient positioning for an orthogonal view, as well as technical nuances throughout the procedure. Using a sequential tubular retractor, they performed a microscopic dissection of the lesion. The tumor was debulked and the tumor capsule was disconnected from the surrounding tissue. During dissection, direct stimulation identified a functional nerve root that was carefully dissected from the tumor capsule. The tumor was then removed en bloc.

The video can be found here: https://stream.cadmore.media/r10.3171/2022.3.FOCVID2220

## Transcript

Mis lateral retroperitoneal transpsoas approach allows gross-total resection of a giant L4 schwannoma.^1^

### 0:28 Case Description.

This is a 60-year-old man referred to my clinic for intermittent tingling and paresthesias involving the dorsum of the right foot, which had significantly worsened over the past 6 months and were affecting his sleep and quality of life.

### 0:41 T1-Weighted Image and Contrast Axial Cuts.

MRI of the lumbar spine showed a contrast-enhancing lesion in the psoas adjacent to the right neuroforamen at L4–5, with remodeling of the foramen, the L4 vertebral body, and L5 superior articular process. Imaging characteristics and clinical presentation were consistent with benign nerve sheath tumor.

### 1:01 Treatment Options.

We discussed treatment options with the patient, including conservative management with imaging follow-up, but due to the progressive symptomatology and tumor size, as well as the need for definitive diagnosis, I favored surgical removal. After discussing the potential surgical approaches, including posterior approach with facetectomy and fusion,^2^ posterior paraspinal approach,^3^ or direct lateral minimally invasive retroperitoneal transpsoas approach,^4^ the patient decided for the lateral approach, which was also my preference.

### 1:30 Patient Positioning.

The patient was taken to the operating room and positioned in the lateral decubitus position. Continuous free-running EMG, direct stimulation EMG, and motor evoked potentials were obtained and remained at baseline throughout the procedure.

### 1:53 Fluoroscopy Identification.

Fluoroscopy was used to localize the level of the L4 pedicle, as well as the anterior and posterior vertebral body line to plan our incision. Meticulous positioning as well as clear orthogonal AP and lateral x-ray views are paramount for this approach to maintain safe working angles to the psoas.

### 1:59 Surgical Approach.

After skin incision, the muscular fascia is identified and sharply incised. Blunt dissection of the external oblique, internal oblique, and transversus abdominus muscle is performed and the transversalis fascia is identified. This is opened bluntly to expose the retroperitoneal fat. Gentle finger dissection is then performed in the retroperitoneal space, and palpation is used to identify the quadratus lumborum, the transverse process, and the psoas. The initial dilator is then placed in a transpsoas fashion using fluoroscopic guidance. The tumor may be palpated as a firm mass within the psoas, and bony changes of the spinal column are helpful for placement of the dilator. EMG stimulation via the dilator is performed to identify location of the lumbar plexus. Sequential dilation is performed, followed by placement of the minimally invasive lateral lumbar retractor, again using fluoroscopic guidance. The Penfield no. 4, along with careful EMG stimulation, is then used for blunt dissection of the superficial psoas, exposing the tumor in the deeper muscle plane. Splitting the muscle in line with its fibers reduces traction on surrounding neural structures.^5,6^

### 3:06 Microscopic Approach.

The surgical microscope is used for tumor dissection. We confirmed no neural activity in the most superficial aspect of the tumor.

### 3:12 Tumor Capsule Opening.

And used the bipolar cautery to open the tumor capsule.

### 3:18 Tumor Debulking.

Tumor forceps are used to obtain samples for frozen section, and we debulked the tumor using aspiration and pituitary forceps. In certain cases, navigation may be used to guide the tumor resection.

### 3:28 Plane Preservation.

The tumor capsule is mobilized, and cotton patties are used to preserve the plane between the capsule and the muscle.

### 3:35 Direct Stimulation EMG.

Direct stimulation is used throughout the procedure before sequential tumor debulking to confirm the absence of neural activity.

### 3:46 Fibrous Tumor Debulking With Ultrasonic Aspirator.

The innermost portion of the tumor was fibrous and resistant to resection, so an ultrasonic aspirator was used to continue tumor debulking. We continued to mobilize and separate the tumor capsule from the surrounding tissue.

### 4:09 Identification of Nerve Root.

A functional nerve root was identified using direct stimulation, and careful dissection was performed to separate it from the tumor capsule.

### 4:37 Hemostasis.

Once the excision was complete, we copiously irrigated the surgical bed and hemostasis was performed. A standard multilayer closure was performed with particular emphasis on closure of the muscle fascia, as lateral muscle wall hernias have been described after this approach.

### 4:53 T1-Weighted Image and Contrast Axial Cuts.

Postoperative MRI showed expected postoperative changes with no evidence of residual tumor.

### 5:00 Postoperative Course.

The patient’s inpatient stay was uneventful. He developed mild (4/5) right hip flexion weakness that did not limit ambulation. We discussed prior to surgery that this could be expected and likely temporary, and this was significantly improved at the 1-month postoperative visit. The patient was discharged home on postoperative day 2 with plans for outpatient rehab.

## Disclosures

Dr. Fox reported other from NuVasive, outside the submitted work.

## Author Contributions

Primary surgeon: Fox. Assistant surgeon: Ravindran. Editing and drafting the video and abstract: Fox, Ramos-Fresnedo, Perez-Vega. Critically revising the work: all authors. Reviewed submitted version of the work: all authors. Approved the final version of the work on behalf of all authors: Fox. Supervision: Fox.
